# Pupil dilation in the Simon task as a marker of conflict processing

**DOI:** 10.3389/fnhum.2013.00215

**Published:** 2013-05-29

**Authors:** Henk van Steenbergen, Guido P. H. Band

**Affiliations:** ^1^Leiden Institute for Brain and CognitionLeiden, Netherlands; ^2^Institute of Psychology, Leiden UniversityLeiden, Netherlands

**Keywords:** aversion, conflict adaptation, conflict processing, cognitive control, effort, emotional arousal, pupil dilation, Simon task

## Abstract

Cognitive demands in response conflict paradigms trigger negative affect and avoidance behavior. However, not all response conflict studies show increases in physiological indices of emotional arousal, such as pupil diameter. In contrast to earlier null-results, this study shows for the first time that small (about 0.02 mm) conflict-related pupil dilation can be observed in a Simon task when stimuli do not introduce a light reflex. Results show that response-conflict in Simon trials induces both pupil dilation and reaction-time costs. Moreover, sequential analyses reveal that pupil dilation mirrors the conflict-adaptation pattern observed in reaction time (RT). Although single-trial regression analyses indicated that pupil dilation is likely to reflect more than one process at the same time, in general our findings imply that pupil dilation can be used as an indirect marker of conflict processing.

## Introduction

When the same environment calls for two incompatible responses, cognitive control is needed to solve the conflict (Botvinick et al., 2001). People tend to minimize cognitive efforts and avoid physical and mental demands such as the need to exert control (Lewin, 1935; Hull, 1943; Winkielman et al., 2003; Morsella et al., 2011). Therefore, conflict typically induces an affective cost, while driving behavioral adjustments to reduce future costs (Botvinick, 2007). Indeed, recent studies have shown that cognitive demands induce negative arousal and trigger avoidance behavior in several tasks (Kool et al., 2010), including response-conflict paradigms (Dreisbach and Fischer, 2012a; Schouppe et al., 2012; Fritz and Dreisbach, 2013; for a review, cf. Dreisbach and Fischer, 2012b). Dreisbach and colleagues (Dreisbach and Fischer, 2012a; Fritz and Dreisbach, 2013) have demonstrated that Stroop conflict triggers negative emotions, and Schouppe and colleagues have shown with a modified Stroop task that conflict triggers avoidance behavior (Schouppe et al., 2012). These results suggest that conflict processing might be characterized by negative affective valence, increased arousal, or both. However, these studies lacked the potential to establish which of the neurocognitive functions conflict processing and affect actually share.

A promising approach to test the putative arousing nature of conflict processing and cognitive control processes is the use of physiological indices of emotional arousal, such as pupil diameter (Bradley et al., 2008; van Steenbergen et al., 2011). Since the seminal work by Hess and Polt (1964) and Kahneman and Beatty (1966; cf. Kahneman, 1973), many pupillometry studies have consistently observed a dilation of the pupil that is, an increase in pupil diameter relative to baseline, in response to increased task demands. This indicates that autonomic arousal as measured by pupil dilation might be an indirect marker of cognitive effort or the costs associated with it (Beatty, 1982; Beatty and Lucero-Wagoner, 2000). In addition, an increasing number of studies have been using pupil dilation as an index of activity in the locus coeruleus-norepinephrine (LC-NE) system (e.g., Jepma and Nieuwenhuis, 2011; Murphy et al., 2011), an arousal-related neurochemical system that is thought to play a crucial role in the cognitive control of behavior (Aston-Jones and Cohen, 2005; Verguts and Notebaert, 2009). Pupil responses have also been observed in more complex conflict tasks such as risky choice from description (e.g., Glöckner et al., 2012). Although pupil dilation effects are larger in errors vs. correct responses (Critchley et al., 2005; Wessel et al., 2011), conflict vs. no-conflict in correct trials has also been shown to increase pupil dilation. This conflict-related pupil dilation has been observed in the Stroop task (Brown et al., 1999; Siegle et al., 2004, 2008; Laeng et al., 2011) and in the flanker task (van Bochove et al., 2013; van Steenbergen et al., unpublished). Other measures of autonomic arousal, including the skin conductance response, have also been reported to be modulated by conflict in the Stroop task (Naccache et al., 2005; Kobayashi et al., 2007).

Taken collectively, these results converge in their support for the hypothesis of a general arousing—and possibly aversive—nature of conflict across different paradigms. However, two recent studies have failed to support a general link between conflict and arousal. Using physiological measures in the context of go/no-go tasks, Schacht and colleagues (Schacht et al., 2009, 2010) showed that the presumed conflict associated with no-go trials (e.g. Band et al., 2003) decreases physiological arousal (Schacht et al., 2009, 2010). Thus, these data speak against the idea that conflict is arousing in the context of no-go trials (see the Discussion for a more elaborate analysis of this issue). More important for the aim of the current paper, Schacht et al. (2010) also reported a null-finding regarding pupil dilation in a Simon task. This null-finding challenges the hypothesis that competition between responses raises physiological arousal. If the absence of a relationship between arousal and conflict would turn out to be a replicable finding, it would limit the validity of prevalent models linking conflict to arousal.

**Figure 1:**
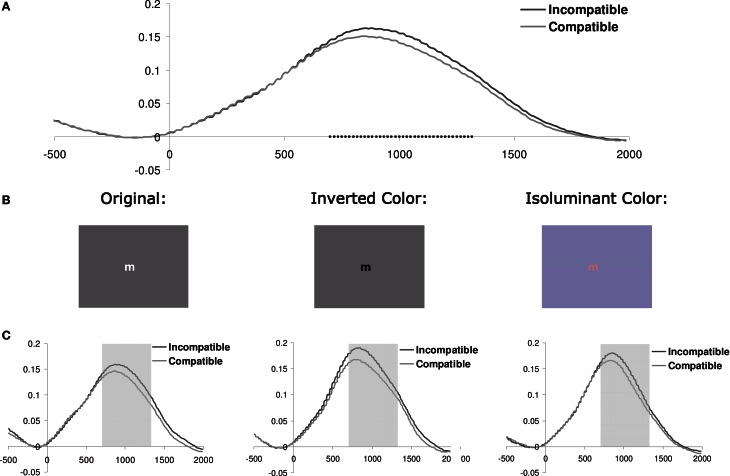
**(A)** Pupil dilation (in mm; relative to a 200 ms prestimulus baseline) for current incompatible vs. compatible trials (pooled across color-scheme conditions) as a function of the onset time (ms) of the stimulus. Squares indicate the samples where paired *t*-tests showed that the difference between incompatible and compatible trials was significant (*p* < 0.05). **(B)** Example of a stimulus used in the three different color-scheme blocks. **(C)** Pupil dilation for each color-scheme. The shaded area shows the interval of interest (700–1300 ms) which was used to calculate the mean pupil dilation (see Figure 2C and Table 1).

The primary goal of the current study was to show that a conflict-related increase in pupil dilation occurs also in a Simon task, provided that one uses a design that is sensitive to the small increase (about 0.02 mm) in pupil diameter that has typically been found for conflict in the Stroop (Brown et al., 1999; Siegle et al., 2004, 2008; Laeng et al., 2011) and the flanker task (van Bochove et al., 2013; van Steenbergen et al., unpublished). One reason why Schacht et al. (2010) may have failed to observe this small effect might be the large (1-mm in amplitude) constriction-dilation complex that was evoked around 300 ms after trial onset in their dataset (see Figure 1A center in Schacht et al., 2010). Pupil constriction and subsequent redilation with this time-course is typically observed in response to changes in ambient lighting that is, when a bright visual stimulus is presented. This well-documented light-reflex response (peak response between 500 and 1000 ms, see Beatty and Lucero-Wagoner, 2000) has been shown to be driven by parasympathetic activity and opposes the influences of sympathetic arousal on the pupil (Loewenfeld, 1993; cf. Steinhauer et al., 2004). Considering the small effects in pupil dilation for Stroop and flanker tasks, it is thus conceivable that the constriction observed by Schacht et al. masked any dilation effect related to conflict in this Simon task. In order to investigate the possibility that this constriction originates from differences in stimulus luminance, we used a Simon task that prevented the introduction of a light reflex by matching the luminance level of the stimuli across the trial (cf. Bradley et al., 2008). We will show that Simon conflict does indeed induce pupil dilation, although we do not observe strong effects driven by differences in stimulus luminance.

The second aim of our study is to analyze pupil dilation in relation to sequential adjustments in behavior as typically observed in the flanker, Stroop, and Simon task (Gratton et al., 1992; Sturmer et al., 2002; Kerns et al., 2004; for a review see Egner, 2007). Sequential effects in these conflict tasks usually show that behavioral interference in a given trial is reduced if it is preceded by a conflict trial. According to the influential conflict monitoring theory (Botvinick et al., 2001), this conflict-adaptation effect might reflect increases in cognitive control that are driven by conflict detected in the preceding trial. Numerous neuroimaging studies have shown that conflict monitoring involves the anterior cingulate cortex (ACC), which is thought to signal the need for additional control to the dorsolateral prefrontal cortex (DLPFC). Similar to evidence from the flanker task (Botvinick et al., 1999) and Stroop task (Kerns et al., 2004), fMRI studies using the Simon task indeed have shown that conflict drives ACC activity (Kerns, 2006; Horga et al., 2011). Consistent with conflict monitoring theory, ACC activity is reduced and DLPFC activity is increased during conflict trials that follow a conflict trial [incompatible-Incompatible (iI) sequence] in comparison to conflict trials that follow a no-conflict trial [compatible-Incompatible (cI) sequence] (Kerns et al., 2004; Kerns, 2006).

Thus, according to the conflict monitoring theory, conflict monitoring and control-related processes can be dissociated because they show an opposite pattern of responding to iI vs. cI trials. Hence, following this logic, we could investigate whether pupil dilation can be used as an indirect marker either of conflict monitoring, or of control. If pupil dilation is smaller for iI than for cI sequences, it might reflect conflict processing (e.g., Siegle et al., 2004); if the reverse pattern is shown, it might be characterized as an index of cognitive control or “effort” (e.g., Kahneman, 1973). If pupil dilation indeed marks conflict processing as opposed to the deployment of control, conflict-related fluctuations in the pupil might also be predictive of subsequent behavioral adaptation. Indeed, such approaches in fMRI studies using the Stroop task (Kerns et al., 2004) and the Simon task (Kerns, 2006; Horga et al., 2011) have revealed that activity in the ACC predicts reduced interference [faster performance in conflict (iI) trials and slower performance in no-conflict (iC) trials] in the subsequent trial.

In sum, the present study has two aims. First, we want to investigate whether the use of an isoluminant trial sequence may result in a conflict-driven increase in pupil dilation for a Simon task. This endeavor also involved testing whether a light reflex and its reversal might be observed if we use the original color-scheme employed by Schacht et al. (2010) vs. a dark (inverse) stimulus color, and whether the presence of such a phenomenon eliminated any conflict-related effect on pupil dilation. Accordingly, our experiment used three different color schemes (original, inverse, isoluminant) which were randomly applied to different blocks of Simon trials. Second, in a first attempt to understand whether pupil dilation in a Simon task reflects conflict- or control-related processes, we performed sequential analyses on this physiological measure. If pupil dilation reflects conflict processing, conflict monitoring theory would predict that it is associated with smaller dilation to iI than cI sequences. Single-trial regression analyses also allowed us to test whether conflict might predict behavioral adjustments in the subsequent trial.

## Methods

### Participants

Thirty-four right-handed university students, with no self-reported history of psychiatric illness and not using medication (except contraception) participated (18–27 years of age; 9 men and 25 women). The experiment was conducted in accordance with relevant regulations and institutional guidelines and was approved by the local ethics committee from the Faculty of Social and Behavioral Sciences. All students read and signed informed consent.

### Simon task

We aimed to replicate the Simon task described by Schacht et al. (2010). Subjects were instructed to make quick and accurate responses to the capital letters “M” and “W” pressing the left or right button press with their thumbs (mapping counterbalanced across subjects) of a mouse they held in their hands. Stimuli were presented using E-Prime® software. Each trial started with a fixation cross presented for 900 ms, after which it changed color (warning cue) for 200 ms. Then the stimulus was presented for 100 ms (plus the after-image duration of the TFT screen) which was followed by a 900-ms blank screen. Responses up to 1000 ms after stimulus presentation were recorded. Stimuli were presented 1.07° randomly to the left or right of the central fixation cross, a location that was either spatially compatible (no-conflict) or spatially incompatible (conflict) with the response participants had to make. Equiprobable conflict and no-conflict trials were randomly presented. Three different color-schemes were applied block-wise (see Figure 1B for examples). The original color-scheme used a dark gray background, a white fixation, a yellow warning cue, and a white stimulus. The inverted color-scheme used the same colors, except for the stimulus color being black. The isoluminant color-scheme was created by using ink colors from the Teufel colors set (Teufel and Wehrhahn, 2000): a slate-blue (RGB-code: 166, 160, 198) background, a dark-cyan (110, 185, 180) fixation, a khaki (188, 175, 81) warning cue, and a salmon (217, 152, 158) stimulus. Using these colors, we approximated isoluminance throughout the whole trial, although this was not photometrically verified.

### Procedure

After informed consent was given, participants were seated in a dimly lit room where the eye tracker was calibrated using Tobii Studio software. Initial eye tracking calibration was used and subsequently verified by graphical feedback using Tobii Studio software. Following a tracking-data quality check (and a repetition of the calibration method if necessary), participants received instructions for the Simon task. They then performed eight practice trials (with accuracy feedback) for all three color-scheme blocks, which were presented in random order. The experiment proper consisted of six blocks of 100 trials each. Each color-scheme was randomly assigned to two out of the six blocks. Following each block, participants received a self-paced break in which the task instructions were repeated.

### Pupil data acquisition and analysis

Pupil diameter was recorded at 60 Hz using a Tobii T120 eye tracker, which is integrated into a 17-inch TFT monitor. Participants were seated at a distance of approximately 60 cm from the monitor. Pupil data were first read into Brain Vision Analyzer using a 60 Hz sampling rate and were then analyzed using custom-made macros. Artifacts including blinks, missing data, and recording problems identified by the eye tracker as invalid data points (about 31% of all the data acquired between −200 and 2000 ms, relative to stimulus onset) were corrected using linear interpolation. A 200-ms pre-stimulus interval was used as baseline for all analyses.

In order to define the interval where pupil dilation was sensitive to the effect of current conflict, we calculated *t*-tests on the difference waves based on the current conflict vs. current no-conflict trials, pooled across all correct trials for all the 34 subjects tested. Trials with less than 50% valid data points available during a −200 to 2000 ms interval were excluded from this analysis. As shown in Figure 1A, conflict effects were observed from 700 to 1300 ms following stimulus onset (*t*-tests thresholded at *p* < 0.05; given that we observed only one cluster of temporally adjacent significantly different samples, correction for multiple comparisons was not applied). Consequently, this interval was used to obtain a measure of mean pupil dilation.

Mean values during baseline (−200 to 0 ms) and dilation intervals (700–1300 ms) were then exported to SPSS, where trials including artifacts that is, unreliably interpolated values (less than 30% valid data points obtained in the baseline interval or the interval of interest), were excluded from subsequent analyses.

Analyses on correct reaction time (RT), error rate, and mean pupil dilation were performed after exclusion of the first trial of each block, trials following an error, and trials that included unreliable pupil-data interpolation in the current or previous trial. As an additional analysis, we also examined the peak of pupil dilation in a 0–2000 ms interval following stimulus onset, for each subject and condition separately. For this analysis the additional trial inclusion criterion was that at least 50% valid data points should have been obtained during this interval.

Greenhouse-Geisser correction was applied when assumptions of sphericity were violated. In these cases, we reported corrected *p*-values and uncorrected degrees of freedom. All significant effects (*p* < 0.05) are reported. MSE and partial eta squared (the proportion of the variance in the dependent variable that is attributable to the respective factor, with other non-error sources of variance being partialled out; cf. Levine and Hullett, 2002) were reported as measures of effect size.

Apart from using ANOVAs with behavioral data (RT and error rate) and pupil dilation (average and peak measures) as dependent variables, we also run an intra-individual correlational analysis that investigated whether mean pupil dilation in a given previous trial predicted RT in the subsequent current trial. In order to do so, we used a regression analysis on individual trials, for each subject and sequential condition separately. Following the method recommended by Lorch and Myers (1990), we then calculated the mean regression coefficient across subjects and used *t*-tests to determine whether regression slopes reliably differed from zero. Because RT and pupil dilation tend to be correlated, we used a step-wise regression approach that allowed us to partial out common variance related to RT in the previous trial, before the predictive effect of previous-trial dilation on the subsequent trial was estimated.

## Results

On initial inspection of the behavioral data, three participants turned out to have made more than 20% errors in one or more of the three color-scheme conditions. In addition, due to technical problems with the eye tracker, pupil data from another seven participants were not available for more than half of the time. These participants were excluded from further analyses, resulting in 24 participants (18–24 years of age; 6 men and 18 women) included in the analyses mentioned below.

### Behavioral data

**Figure 2:**
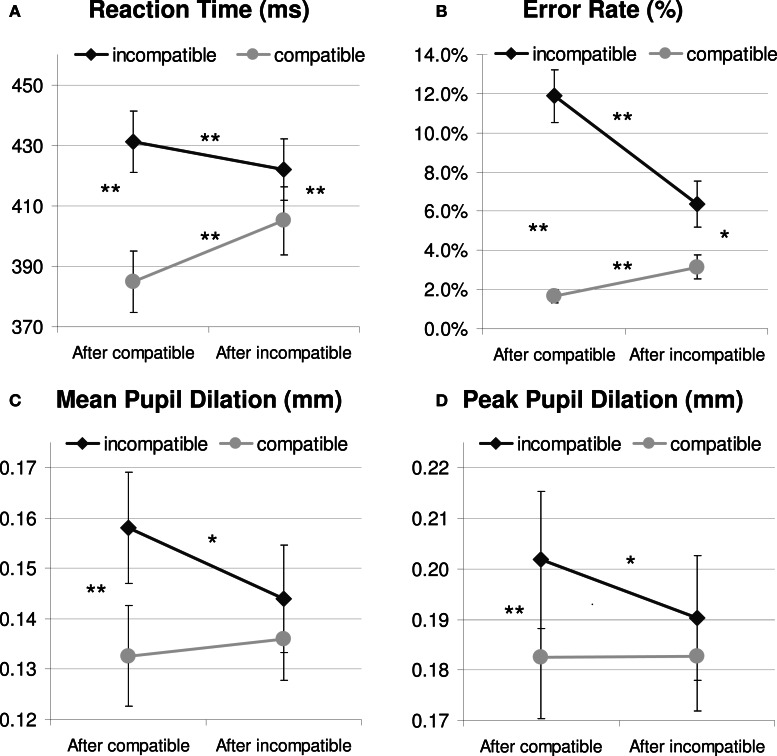
**Behavioral (A and B) and pupil dilation data relative to a 200 ms prestimulus baseline (C and D) as a function of current and previous compatibility.** ^*^p <.05; ^**^p < 0.01.

As Figure 2A and Table 1 show, the Simon task produced standard response conflict and sequential adaptation effects in RT. These were revealed by a 2 × 2 repeated-measures ANOVA showing a significant main effect of current compatibility [*F*_(1, 23)_ = 214.1, *p* < 0.001, MSE = 337.289, η^2^_*p*_ = 0.903] and a significant interaction effect between current and previous compatibility [*F*_(1, 23)_ = 81.5, *p* < 0.001, MSE = 191.627, η^2^_*p*_ = 0.780]. Thus, incompatible trials produced longer RTs than compatible trials, and this interference effect was reduced if there was conflict in the previous trial, see Figure 2A. There was also a reliable main effect of previous compatibility, showing post-conflict slowing in performance [*F*_(1, 23)_ = 7.2, *p* = 0.013, MSE = 317.78, η^2^_*p*_ = 0.238]. *Post-hoc* tests showed that iI trials were faster than cI trials, *t*_(23)_ = −3.28, *p* < 0.005, whereas iC trials were slower than cC trials, *t*_(23)_ = 8.01, *p* < 0.001. These effects occurred independently of the color-scheme used (*F*s < 1.1, see Table 1 for details). However, color-scheme did have a main effect on absolute RT [*F*_(2, 46)_ = 44.4, *p* < 0.001, MSE = 1165.434, η^2^_*p*_ = 0.659], showing slower RTs for the isoluminant condition (435 ms) than for the other original (399 ms) and matched conditions (399 ms). This is likely due to the reduced contrast between the isoluminant stimulus and background which impairs the identification of the target.

**Table 1:** **Behavioral and pupil dilation data for each condition**.

**Color scheme and condition**	**Artifacts**			**Behavioral data**		**Baselined pupil dilation (mm)**
	**No. of trials with/**	**without/**	**proportion (%)**	**RT (ms)**	**Error rate (%)**	**Mean 700–1300 ms**
**ORIGINAL**						
cC	44	31	29.0	372	1.5	0.133
cl	46	33	29.3	420	10.5	0.156
iC	47	34	28.1	394	3.4	0.121
il	39	28	28.3	410	5.0	0.129
Average	44	31	28.7	399	5.1	0.135
Conflict effect	−3	−2	0.3	32	5.3	0.015
Conflict-adaptation effect	11	8	0.1	32	7.4	0.015
**INVERTED**						
cC	43	31	28.6	375	1.9	0.134
cl	45	32	28.9	417	13.1	0.163
iC	47	34	27.8	394	3.2	0.151
il	38	26	30.5	408	7.4	0.162
Average	43	31	28.9	399	6.4	0.153
Conflict effect	−3	−3	1.5	28	7.7	0.020
Conflict-adaptation effect	11	8	−2.4	28	6.9	0.017
**ISOLUMINANT**						
cC	43	34	21.3	407	1.5	0.130
cl	46	37	20.3	456	12.0	0.155
iC	47	37	20.4	428	2.8	0.136
il	38	30	20.9	448	6.6	0.141
Average	44	34	20.7	435	5.8	0.141
Conflict effect	−3	−2	−0.2	35	7.1	0.015
Conflict-adaptation effect	12	10	−1.5	29	6.7	0.019

*Note: Table displays averages across participants. Conflict effect, (([iI] + [cI]) − ([iC] + [cC]))/2; conflict-adaptation effect, ([cI] − [cC]) − ([iI] − [iC]); proportion artifacts, (No. of trials with artifacts – No. trials without artifacts)/No. of trials with artifacts. Behavioral and pupil dilation (relative to a 200 ms prestimulus baseline) data are based on the trials without artifacts*.

An analysis on error rate (see Table 1 and Figure 2B) mirrored the RT data and revealed significant effects of current compatibility [*F*_(1, 23)_ = 41.6, *p* < 0.001, MSE = 0.008, η^2^_*p*_ = 0.644], previous compatibility [*F*_(1, 23)_ = 17.6, *p* < 0.001, MSE = 0.002, η^2^_*p*_ = 0.433] and their interaction [*F*_(1, 23)_ = 25.6, *p* < 0.001, MSE = 0.003, η^2^_*p*_ = 0.527]. Conflict and sequential adaptation effects were in the same direction as the RT data. However, the effect of previous conflict was opposite to the post-conflict slowing observed in RT: participants made less errors after incompatible (4.8%) than after compatible trials (6.8 %). This speed-accuracy trade-off suggests that apart from improving control, conflict also induced a more conservative way of responding in this task.^1^

Given that some authors have advocated the exclusion of complete stimulus repetitions in order to correct for lower-order priming effects (Mayr et al., 2003; cf. Egner, 2007), we repeated this analysis on data that excluded complete stimulus repetitions (i.e., when two consecutive Simon trials present the same stimulus at the same location). This analysis yielded a very similar pattern of results that showed the same effects being significant.

### Mean pupil dilation

Baseline-corrected pupil diameter change in response to the stimulus is plotted for each color-scheme in Figure 1C. In line with our prediction, visual inspection suggested that pupil diameter is increased for current incompatible in comparison to current compatible Simon trials, an effect that appears to be independent of the particular color-scheme used. Thus, contrary to the previous report, dilation following incompatible stimuli was also shown in the condition that used the original color-scheme employed by Schacht et al. (2010). Interestingly, we did not observe the constriction-dilation complex following stimulus onset that was reported by these authors. Note that the inverse color-scheme condition only slightly increases the overall dilation in comparison to the original color-scheme, which suggests that the luminosity difference between the dark and light stimulus in this task only induces subtle changes in pupil diameter.

Baselined mean dilation (700–1300 ms) was subsequently analyzed using repeated-measures ANOVAs. Table 1 describes the results from this analysis and also shows the mean number of trials before and after artifact rejection for all conditions. Analyses on the proportion of artifact trials (see Table 1) only revealed a significant effect of color-scheme, *F*_(2, 46)_ = 5.0, *p* = 0.016, MSE = 0.05, η^2^_*p*_ = 0.178, showing a reduced proportion of artifacts in the isoluminant condition.

Analyses on mean dilation (700–1300 ms) confirmed that Simon incompatibility increased pupil dilation [*F*_(1, 23)_ = 15.6, *p* = 0.001, MSE = 0.001, η^2^_*p*_ = 0.404; see Table 1 for details]. Mirroring the behavioral pattern, there was also an interaction between current and previous compatibility [*F*_(1, 23)_ = 4.4, *p* = 0.047, MSE = 0.001, η^2^_*p*_ = 0.161], see Figure 2C. In line with the hypothesis that pupil dilation reflects conflict processing, *post-hoc* tests showed that iI trials induced less dilation than cI trials, *t*_(23)_ = −2.43, *p* = 0.023, whereas iC trials were not significantly different from cC trials, *t*_(23)_ = 0.52, *p* = 0.606. In contrast to the behavioral data, there was no main effect of previous compatibility [*F*_(1, 23)_ = 1.5, *p* = 0.239, MSE = 0.001, η^2^_*p*_ = 0.060], although a significant color-scheme × previous compatibility interaction [*F*_(2, 46)_ = 4.3, *p* = 0.026, MSE = 0.001, η^2^_*p*_ = 0.158] revealed a confound introduced by differences in stimulus luminance. *Post-hoc* tests revealed a post-conflict decrease in dilation for the original color-scheme, *M* = −0.0199 mm, *t*_(23)_ = −2.77, *p* = 0.011. There was no significant post-conflict effect for the inverted color-scheme, *M* = 0.0077 mm, *t*_(23)_ = 0.93, *p* = 0.363 and the isoluminant color-scheme *M* = −0.0040 mm, *t*_(23)_ = −0.76, *p* = 0.455. Color-scheme condition did not interact with other (combinations of) conditions (*F*s < 1.5), and a main effect that would be suggestive of overall differences in pupil diameter did not reach significance [*F*_(2, 46)_ = 2.1, *p* = 0.131, MSE = 0.004, η^2^_*p*_ = 0.085].

Analyses were repeated on pupil data that excluded complete stimulus repetitions. This repeated-measures ANOVA produced the same effects, except for the interaction between previous and current compatibility not reaching significance, *F*_(1, 23)_ = 1.2, *p* = 0.285, MSE = 0.002, η^2^_*p*_ = 0.050. However, given that this analysis reduces the original number of trials for the cC and iI conditions by 50%, it is likely that this analysis lacked statistical power to detect this effect. Separate *t*-test still confirmed a conflict-adaptation pattern: robust interference effects in pupil dilation were observed after compatible trials [*t*_(23)_ = 3.93, *p* < 0.001], but not after incompatible trials [*t*_(23)_ = 1.63, *p* = 0.117].

### Additional analysis: peak pupil dilation

To test for the robustness of our findings, we also used an alternative way of analyzing pupil dilation by extracting peak values of dilation.^2^ Peak detection was carried out on the individual baseline-corrected averages for all combinations of sequential conditions and all six blocks separately. Given that each of the three color schemes was presented twice during the experiment, we could thus control for potential time and order effects which could have affected the latency of the pupil dilation response and the estimated pupil dilation when measured over a fixed time interval.

Data from the 22 participants who had at least five trials per condition were analyzed. Table 2 describes the peak amplitude and latency as well as the mean number of trials before and after artifact rejection for all conditions. Analyses on the proportion of artifacts only revealed a significant effect of color-scheme, *F*_(2, 42)_ = 3.7, *p* = 0.034, MSE = 0.048, η^2^_*p*_ = 0.151, showing a reduced proportion of artifacts in the isoluminant condition.

**Table 2:** **Peak pupil dilation data for each condition and separate for block sequence**.

**Color scheme and condition**	**First block**					**Second block**				
	**Artifacts**			**Baselined peak pupil dilation**		**Artifacts**			**Baselined peak pupil dilation**	
	**No. of trials with/**	**without/**	**proportion (%)**	**Latency (ms)**	**Amplitude (mm)**	**No. of trials with/**	**without/**	**proportion (%)**	**Latency (ms)**	**Amplitude (mm)**
**ORIGINAL**										
cC	22	16	26.3	936	0.185	22	16	26.9	875	0.173
cl	23	17	26.1	902	0.181	23	16	28.1	982	0.198
IC	24	18	24.4	946	0.174	23	17	25.3	848	0.155
il	20	14	26.6	874	0.168	19	15	24.0	893	0.174
Average	22	17	25.8	915	0.177	22	16	26.1	899	0.175
Conflict effect	−2	−1	1.0	−53	−0.005	−1	−1	−0.1	76	0.022
Conflict-adaptation effect	6	5	−2.4	37	0.002	5	3	2.8	61	0.007
**INVERTED**										
cC	22	17	22.6	870	0.209	21	15	30.7	867	0.161
cl	22	17	24.2	874	0.220	23	16	30.7	858	0.204
IC	23	18	23.0	893	0.194	23	16	31.6	882	0.205
il	19	14	28.1	864	0.216	19	13	29.3	857	0.205
Average	22	16	24.5	875	0.210	22	15	30.6	866	0.194
Conflict effect	−2	−2	3.3	−12	0.016	−1	−1	−1.2	−17	0.022
Conflict-adaptation effect	5	4	−3.5	33	−0.011	5	3	2.3	17	0.043
**ISOLUMINANT**										
cC	21	17	21.8	864	0.178	21	16	22.5	839	0.189
cl	24	19	20.6	873	0.214	22	17	22.4	827	0.193
iC	24	19	19.3	898	0.189	23	18	22.3	841	0.180
il	20	16	18.3	866	0.195	18	14	24.8	864	0.185
Average	22	18	20.0	875	0.194	21	16	23.0	842	0.187
Conflict effect	−1	0	−1.1	−12	0.021	−2	−2	1.2	5	0.004
Conflict-adaptation effect	6	5	−0.2	40	0.030	6	5	−2.6	−35	−0.001

*Note: Table displays averages across participants. Conflict effect, (([iI] + [cI]) − ([iC] + [cC]))/2; conflict-adaptation effect, ([cI] − [cC]) − ([iI] − [iC]); proportion artifacts, (No. of trials with artifacts – No. of trials without artifacts) / No. of trials with artifacts. Pupil dilation (relative to a 200 ms prestimulus baseline) data are based on the trials without artifacts*.

Note that numerical conflict and conflict-adaptation effects on peak dilation amplitude were not reliably observed in all six blocks, likely because the low number of trials included in each block introduced noise (see Table 2). Peak amplitude measures are well-known to be more sensitive to noise than mean amplitude measures. That is why mean amplitude is the preferred measure, at least in ERP research (Woodman, 2010). Nonetheless, a repeated measures ANOVA revealed that current conflict reliably increased pupil amplitude across blocks, *F*_(1, 21)_ = 12.9, *p* = 0.002, MSE = 0.002, η^2^_*p*_ = 0.380. Again, there was no main effect of previous compatibility *F*_(1, 21)_ = 1.9, *p* = 0.188, MSE = 0.002, η^2^_*p*_ = 0.081, and there was a trend for a significant color-scheme × previous compatibility interaction *F*_(2, 42)_ = 3.3, *p* = 0.056, MSE = 0.002, η^2^_*p*_ = 0.137. This interaction again revealed a confound introduced by differences in stimulus luminance. *Post-hoc* tests revealed a post-conflict decrease in dilation for the original color-scheme, *M* = −0.0165 mm, *t*_(21)_ = −2.57, *p* = 0.018. There was no significant post-conflict effect for the inverted color-scheme, *M* = 0.0062 mm, *t*_(21)_ = 0.80, *p* = 0.434 and the isoluminant color-scheme *M* = −0.0064 mm, *t*_(21)_ = −1.23, *p* = 0.234. Notably, the peak analysis did not show a significant interaction between current and previous compatibility *F*_(1, 21)_ = 2.6, *p* = 0.124, MSE = 0.002, η^2^_*p*_ = 0.109, although this might be (partly) due to reduced power and increased noise introduced by the peak detection method. Separate *t*-tests still confirmed a conflict-adaptation pattern: robust interference effects in peak dilation were observed after compatible trials *t*_(21)_ = 4.29, *p* < 0.001, but not after incompatible trials *t*_(21)_ = 1.29, *p* = 0.210. See Figure 2D for details.

Corroborating the validity of the mean dilation analysis described earlier, there was no evidence for an interaction effect of current and previous conflict on the latency of the peak, *F*_(1, 21)_ = 1.2, *p* = 0.288, MSE = 18210.9, η^2^_*p*_ = 0.054. The analysis on peak latency revealed a main effect of color-scheme, *F*_(2, 42)_ = 4.5, *p* = 0.021, MSE = 27253.7, η^2^_*p*_ = 0.175, a main effect of sequence *F*_(1, 21)_ = 4.9, *p* = 0.038, MSE = 9818.0, η^2^_*p*_ = 0.190, and an interaction effect between color-scheme, sequence, and current conflict *F*_(2, 42)_ = 5.1, *p* = 0.021, MSE = 15637.2, η^2^_*p*_ = 0.196. See Table 2 for details.

### Regression analysis: pupil dilation predicting subsequent behavioral adaptation

If pupil dilation marks conflict-related signaling (presumably originating from the ACC) that drives improved cognitive control, it should be predictive of faster responses on subsequent conflict and slower responses on subsequent no-conflict trials (cf. Kerns, 2006; Horga et al., 2011). To test this hypothesis, we used a regression analysis on individual trials. This analysis tested the predictive effect of Trial *N* − 1 baselined mean pupil dilation on subsequent behavioral performance in Trial *N*, after common variance related to behavioral performance in Trial *N* − 1 was partialled out as a first step. Given that the sequential analysis revealed previous-conflict effects on pupil dilation for the non-matched color-scheme conditions, the current analysis only included data from the isoluminant condition.

If pupil dilation in Trial *N*−1 predicts control improvement in Trial *N*, the regression analysis should reveal a negative coefficient when analyzing iI trials (incompatible-trial RTs decrease after conflict) and a positive regression coefficient when analyzing iC trials (compatible-trial RTs increase after conflict). Contrary to this prediction, negative slopes tend not only to be observed for the iI sequence [*t*_(23)_ = −1.78, *p*_1-sided_ = 0.044; after stimulus-repetition exclusion: *t*_(23)_ = −2.22, *p*_1-sided_ = 0.019], but also for the iC sequence [*t*_(23)_ = −2.31, *p* = 0.030]. For reasons of completeness, we also analyzed cC and cI trials. Coefficients for the cC sequence [*t*_(23)_ = −1.81, *p* = 0.084; after stimulus-repetition exclusion: *t*_(23)_ = −1.24, *p* = 0.241] and the cI sequence [*t*_(23)_ = −0.49, *p* = 0.632] were not consistently different from zero. Thus, pupil dilation in Trial *N*−1 tends to predict overall faster responding—but not increased attentional control—in Trial *N* for previous conflict trials only.

## Discussion

Using pupillometry, this study shows for the first time that response conflict in a Simon task accompanies changes in physiological arousal. Unlike an earlier published null-finding (Schacht et al., 2010), our findings do reveal increased pupil dilation to response conflict in a Simon task. These effects were independent of whether the block of trials used isoluminant or mismatched color schemes. In addition, we are the first to show that sequential analyses reveal adjustments in behavior and pupil dilation that are consistent with the notion that pupil dilation can be used as a marker of conflict-related processing. However, regression analyses on single-trial data showed that pupil dilation might also reflect processes other than response-conflict monitoring.

The pupil dilation increase to conflict we observed in the Simon task extends earlier findings that have shown similar dilation effects in other response-conflict tasks including the Stroop task (Brown et al., 1999; Siegle et al., 2004, 2008; Laeng et al., 2011) and the flanker task van (van Bochove et al., 2013; van Steenbergen et al., unpublished). As in those studies, we observed that the magnitude of this effect is quite small (i.e., in the order of 0.02 mm). Interestingly, our data did not show any evidence for a constriction due to a light-reflex response, masking conflict-driven pupil dilation effects. Thus, in contrast to Schacht et al. (2010), we did observe conflict-driven pupil dilation, even in the condition that used their original color scheme. Apparently, the small size and/or the short presentation of the target (100 ms) was not sufficient to elicit this typical light reflex (Beatty and Lucero-Wagoner, 2000) observed in other studies using larger stimulus displays and longer presentation times (e.g., Bradley et al., 2008). However, given that our study closely replicated the trial structure reported by Schacht et al. (2010) which did observe a light reflex using the same stimulus duration, other factors may explain why a constriction-dilation complex was observed in this previous report but not in our dataset. Possibly, procedural differences are responsible for this effect and the null-finding obtained in the previous report^3^.

Apart from presenting the finding that response conflict in a Simon task increases pupil dilation, the additional analyses described novel findings that further contribute to the recent debate about the aversive and arousing nature of conflict (Botvinick, 2007; Schacht et al., 2009, 2010) and how affect might contribute to adjustments in cognitive control. We showed that the standard sequential effects observed in behavior were also observed in the pupil dilation response. In particular, iI trials were faster and showed less pupil dilation than cI trials (see Figure 2). However, the difference observed for behavior in iC vs. cC trials, was not observed in pupil dilation. A similar pattern of results has been observed in neuroimaging studies focusing on the ACC (e.g., Botvinick et al., 1999; Kerns et al., 2004; Kerns, 2006), suggesting that the ACC works as a conflict monitor (but see also Brown, 2011; Grinband et al., 2011; Yeung et al., 2011). In line with conflict monitoring theory logic, the pupil dilation response might thus reflect conflict monitoring rather than control implementation processes. This conclusion also concords with neuroscientific studies revealing the ACC to be a key generator of autonomic arousal responses (Critchley et al., 2003, 2005; Critchley, 2005).

It is interesting to note that these physiological effects in pupil dilation and ACC divert from our behavioral effect showing that current congruent trials appeared to be more sensitive to sequential effects than incongruent trials. Some authors have argued that such behavioral effects are at odds with conflict monitoring theory and might reflect the operation of a general context adaptation mechanism rather than a mechanism that specifically responds to conflict (Schlaghecken and Martini, 2012). It is also possible that those effects are (partly) driven by post-conflict slowing effects (Verguts et al., 2011) which might mask the sequential effects on incongruent trials. Although more research is needed to understand the processes responsible for our behavioral results, the current study suggests that pupil dilation differences might most closely reflect conflict-monitoring related processes.

The observation that physiological arousal mirrors the trial-to-trial modulation observed for conflict processing is also consistent with claims that the affective quality of conflict processing might play a functional role in driving adjustments in behavior (Botvinick, 2007; cf. Proulx et al., 2012). In addition to the role that arousal is shown to play, several recent studies have suggested that negative affect and positive affect, respectively, augments and decreases conflict-driven behavioral adjustments in the flanker (van Steenbergen et al., 2009, 2010, 2012a; cf. Kuhbandner and Zehetleitner, 2011) and the Simon task (van Steenbergen et al., 2012b). These findings suggest that affect-congruent processing of the putative aversive conflict may facilitate conflict adaptation (but see also Padmala et al., 2011). Other studies using performance-contingent reward have shown evidence for motivational effects on conflict adaptation as well (Padmala et al., 2011; Sturmer et al., 2011; Braem et al., 2012). Taken together, these results suggest that the aversive or motivational arousing aspect of conflict might drive subsequent cognitive control. Pupil dilation analyses as carried out in this manuscript might be useful in future studies to further investigate those effects.

It is important to note that it is currently unknown whether arousal in itself is sufficient to drive control adaptations, or whether only specific types of emotional arousal—such as those combined with a negative valence (cf. Thayer's (1989) conception of “tense arousal”)—will produce these effects (cf. van Steenbergen et al., 2011). In particular, further investigation is warranted because facial corrugator muscle activity, a physiological marker of negative valence, has been shown to respond to aversive stimulation (e.g., Larsen et al., 2003) and cognitive and physical effort (e.g., Cacioppo et al., 1985; Boxtel and Jessurun, 1993; de Morree and Marcora, 2010), whereas it was shown to be insensitive to conflict as measured in the Simon task (Schacht et al., 2010). It is important to note that Schacht et al. (2009, 2010) did demonstrate conflict-driven increases in the corrugator muscle during a go-nogo task which accompanied arousal-related decreases as measured with pupil dilation and skin conductance responses. Such evidence suggests that not all types of conflicts are equally aversive and arousing, and/or that a comparison between nogo and go trials introduces factors other than conflict (such as different motor affordances, cf. Fritz and Dreisbach, 2013) that might affect physiological measures.

However, it is important to note that the results from the single-trial regression analyses do not allow us to conclude that pupil dilation is a pure measure of conflict processing: conflict-induced pupil dilation (during a previous incompatible trial) tended to predict increased speed of subsequent responding to both incompatible and compatible trials. This finding is inconsistent with the interpretation that pupil dilation is a pure index of conflict processing, which—following conflict-monitoring logic—would have predicted increased attentional control, thus speeding up performance on incompatible relative to compatible trials. These results emphasize that pupil dilation might reflect more than one process, rather than being a process-pure measure of conflict monitoring. Part of the variance in pupil diameter might reflect other processes, such as anticipatory effort or motor preparation (cf. Cohen et al., 2000). Such processes might be related to elevated levels of task-induced arousal, which might facilitate a subsequent general speed-up in responding. Neuroimaging studies might determine whether such processes reflect activity in different regions within or beyond the ACC (cf. Critchley et al., 2005).

We mention two limitations of the current study. First, even though behavioral conflict-adaptation effects were quite large (around 30 ms) and robust, the sequential effects obtained in pupil dilation were less robust, in spite of the many trial repetitions used. Indeed, when using a peak measure of dilation, an approach known to be susceptible to noise, the interaction between previous and current conflict failed to reach statistical significance. Second, based on the sequential effects obtained, we argued that pupil dilation most likely reflects conflict processing indicating the need for additional control, rather than reflecting control processes that help to overcome conflict. However, this reasoning critically hinges on the central assumption of conflict monitoring theory that conflict-driven control adjustments are implemented across trials that is, that they only start on the subsequent trial (Botvinick et al., 2001). In contrast, recent data (Scherbaum et al., 2011) and modeling work (Scherbaum et al., 2012) have suggested that control adjustment might already start within the trial *N*−1 itself. According to these findings, sequential effects result from a carry over of control settings in trial *N*, which have already been adapted during trial *N*−1. This and other recent discussions concerning the link between conflict-monitoring activity and time-on-task effects (Brown, 2011; Grinband et al., 2011; Yeung et al., 2011) emphasize that future studies are needed to further understand and dissociate conflict and control processes and their temporal dynamics.

In conclusion, our findings show that response conflict in Simon trials increase behavioral costs measured in RT and pupil dilation. Sequential analyses also showed that pupil dilation mirrored the pattern observed in RTs. Along with previous studies, these findings show that pupil diameter might be used as an indirect marker of conflict monitoring. We hope these findings encourage researchers to use pupil dilation in future studies. Such studies are needed to further our understanding of the role that emotional arousal plays in driving and modulating conflict-driven control.

### Conflict of interest statement

The authors declare that the research was conducted in the absence of any commercial or financial relationships that could be construed as a potential conflict of interest.

## Footnotes

^1^To evaluate the consequences of data loss, we also ran ANOVAs on the behavioral data including the seven participants with eyetracker recording problems. The only difference in the performance analyses was an additional effect of color-scheme, *F*_(2, 60)_ = 3.7, *p* = 0.033, MSE = 0.002, η^2^_*p*_ = 0.110, showing that participants made slightly more errors in the context of the inverted color scheme (7.3%; original: 6.0%; isoluminant: 6.5%).

^2^ We thank an anonymous reviewer for this suggestion.

^3^It could be possible that ambient lighting conditions in the current study prevented us from observing a light reflex. In addition, differences in the acquisition hardware or analysis of the pupil data might be responsible. For example, it could be speculated that the analysis in the previous report might have been less powerful since it discarded all segments including blinks and artifacts. When we excluded all trials including artifacts in a period from −1000 to 2500 ms and analyzed correct incompatible and compatible trials pooled across color-scheme conditions, only 25 of the original 34 participants had trials available for analysis (mean number of trials for congruent and incongruent conditions 25 and 23, respectively). There was no evidence for any conflict-induced pupil dilation in this dataset. The analysis described in the main text of this paper used linear interpolation which allows for the inclusion of more trials in the analysis.
